# The novel protein C9orf116 promotes rat liver cell line BRL-3A proliferation

**DOI:** 10.1371/journal.pone.0180607

**Published:** 2017-07-27

**Authors:** Chunyan Zhang, Cuifang Chang, Weiming Zhao, Hang Gao, Qiwen Wang, Deming Li, Fuchun Zhang, Shifu Zhang, Cunshuan Xu

**Affiliations:** 1 Xinjiang Key Laboratory of Biological Resources and Genetic Engineering, College of Life Science and Technology, Xinjiang University, Urumqi, China; 2 State Key Laboratory Cultivation Base for Cell Differentiation Regulation, College of Life Science, Henan Normal University, Xinxiang, Henan, China; 3 Henan Engineering Laboratory for Bioengineering and Drug Development, College of Life Science, Henan Normal University, Xinxiang, Henan, China; Duke University School of Medicine, UNITED STATES

## Abstract

Our previous study has proved that the chromosome 9 open reading frame 116 (C9orf116) (**NM_001106564.1**) was significantly up-regulated in the proliferation phase of liver regeneration. To study its possible physiological function, we analyzed the effect of C9orf116 on BRL-3A cells via over-expression and interference technique. MTT results showed that the cell viability of the interference group was significantly lower than the control group at 48h after transfection (*P*<0.05), whereas it was significantly higher in the over-expression group (*P*<0.05). The flow cytometry results showed that C9orf116 knockdown or over-expression had little effect on BRL-3A cell apoptosis. However, the number of cells in division phase (G2/M) was significantly reduced in the interference group (*P*<0.05), but significantly increased in the over-expression group (*P*<0.01). Furthermore, the expressions of cell proliferation-related genes CCNA2, CCND1 and MYC both at mRNA and protein levels were down-regulated in the interference group and up-regulated in the over-expression group. Therefore, we concluded that C9orf116 may promote cell proliferation by modulating cell cycle transition and the expression of key genes CCNA2, CCND1 and MYC in BRL-3A cells.

## Introduction

Liver is an organ with extraordinary ability to regenerate. After liver injury or partial hepatectomy (PH), the remnant liver tissue can regenerate quickly to restore the original size and weight, to maintain optimum liver weight/body weight ratio, and ultimately to rebuild liver tissue structure and liver function, which is known as liver regeneration (LR) [1, 2]. LR was divided into three key stages [3–6]: the initiation stage (2-6h), in which quiescent hepatocytes were activated and G0/G1 conversion occurred; the proliferative stage or advanced stage (12-72h), in which hepatocytes proliferation occurred; the termination stage (120-168h), in which liver regeneration terminates with the rebuilding of structure and function of tissue [7]. LR involves cell activation, differentiation, proliferation and regulation, re-differentiation, tissue structure and function reconstruction and other physiological activities [1, 8–10]. It is well known that multiple-growth factors, hormones, and other bioactive molecules are involved in the LR through a variety of signaling pathways. Our previous study found C9orf116 was significantly up-regulated in LR proliferation stage [11, 12]. Therefore, we hypothesized that C9orf116 may regulate the proliferation of liver cells.

The full-length of the chromosome 9 open reading frame 116 (C9orf116) is 4351 bp, containing three exons and two introns. The three exons are located 1–211, 951–1029, 3764–4351 bp. The full length of mRNA(**NM_001106564.1**) is 878 bp, encoding 136 amino acids. The sequences of three homologous genes of rat, mouse and human have similar structures and almost the same length of the CDS. The similarity of amino acid sequences reaches 90.74% among rats, mouse and human. Three homology proteins share a conserved domain of DUF4490 with unknown function. This domain family was first discovered in eukaryotes. The DUF4490 domain is typically 101–220 amino acids in length. In mice, the members of the family could be induced by p53, and played roles in DNA damage response which suggests they may be involved the maintaining of genomic integrity against genotoxic stresses, such as UVC irradiation [13]. However, it was unclear if C9orf116 can also promote the proliferation of cultured rat liver cells. Therefore, we used BRL-3A rat liver cell to study the function of C9orf116 *in vitro* by adding and knocking down genes technologies. The results showed that when BRL-3A cells were treated with C9-siRNA, the cell viability was decreased and the proportion of cells in G2/M was reduced. In this process the expression of cell proliferation-related genes including CCNA2, CCND1 and MYC was also reduced at both mRNA and protein levels. However, the opposite effects were observed when BRL-3A cells have over-expressed C9orf116. Based on these data, we hypothesized that C9orf116 promotes rat liver cell line BRL-3A proliferation by modulating cell cycle transition and the expression of key genes CCNA2, CCND1 and MYC in BRL-3A cells.

## Materials and methods

### Cell culture

Human embryo kidney cells 293T and rat liver cell line BRL-3A were obtained from cell bank of the School of Basic Medicine of Peking Union Medical College (China). Cells were cultured in Dulbecco’s modified Eagle’s medium (DMEM, Life technologies, USA) supplemented with 10% fetal bovine serum (Gibco) and 1% penicillin/streptomycin at 37°C in a 5% CO2 incubator with saturated humidity.

### Synthesis of siRNA targeting C9orf116 and RNA interference

The siRNAs targeting C9orf116(C9-siR1,2,3) and their negative control (NC) were obtained from Ribobio (Guangzhou, China) (Table 1). BRL-3A cells were transfected with the indicated siRNA (50 nM) using Lipofectamine RNAiMAX (Invitrogen, USA) according to manufacturer's instruction. The expression change of C9orf116 was determined by RT-PCR at 48 h after transfection.

**Table 1:** The sequence of C9orf116 siRNAs.

siRNA	Target sequence	Sequence
*C9orf116* siR1	GAATGTTCCGGAGACATAA	5′GAAUGUUCCGGAGACAUAAdTdT3′
		3′dTdTCUUACAAGGCCUCUGUAUU5′
*C9orf116* siR2	GCATGAGATGCCGAAAGCA	5′GCAUGAGAUGCCGAAAGCAdTdT3′
		3′dTdTCGUACUCUACGGCUUUCGU5′
*C9orf116* siR3	CCAATGAAGCTGTTTCCAT	5′CCAAUGAAGCUGUUUCCAUdTdT3′
		3′dTdTGGUUACUUCGACAAAGGUA5′

### Plasmid construction and lentivirus production

Coding sequence of rat C9orf116 (**NM_001106564.1**) was synthesized and inserted into the multiple cloning site (MCS) of the lentiviral vector pCDH-CMV-MCS-EF1-copGFP by Generay Biotech (Shanghai, China). Vector particles were produced in HEK293T cells by transient cotransfection involving a three-plasmid expression system. Viral packaging was processed according to Dai *et al* [14] and Ding *et al* [15]. The concentrated virus particles were suspended in PBS and stored at -80°C.

### Transduction of BRL-3A

Transduction was performed in 24-well plates. BRL-3A cells were seeded at 1 × 10^5^ cells per well. One day later, the cells were transduced with 2 × 10^5^ TU virus particles of C9orf116 for 8 h and the viral infection was serially repeated 2–3 times. After three days post the last round of transduction, the efficiency was measured by detecting GFP fluorescent protein using fluorescence microscope. After 1 or 2 weeks, transduced cells in clusters were partially digested and seeded into new dishes to continue their culture.

### RNA isolation and quantitative RT-PCR analysis

Total cellular RNA was extracted using Trizol (Invitrogen Corporation, Carlsbad, California, USA) according to the manufacturer’s instructions. The integrity of RNA was determined by denaturing agarose gel electrophoresis (70 v, 20 min). RNA purity was analyzed by spectrophotometer at 260 nm and 280 nm absorbance value (A260/280). Qualified RNA (2 μg) was used to synthesize the first strand of cDNA following the reverse transcription kit (Promega,USA). Gene expression was determined by Quantitative real-time PCR (qRT-PCR) using a SYBR Green master mix kit (Qiagen, Germany) according to the manufacturer’s protocol. QRT-PCR was performed using SYBR® Green I on a Rotor-Gene 3000 real-time analyzer (Corbett Robotics, Brisbane, Australia) as described previously [16]. The primers were synthesized by Shanghai Generay Biotech Co, Ltd and listed in Table 2. Each sample was analyzed in triplicate. GAPDH was used as internal control for the normalization of total mRNA in each sample. The relative expression of target genes was calculated with the 2-ΔΔCt method.

**Table 2:** The primer sequences used in the RT-PCR.

Genes	Forward primer	Reverse primer
*C9orf116 (***NM_001106564.1**)	5'-GCCAAATGTCTGAAGAAAACC-3'	5'-GTACTGTAGCCATGAAACCACC-3'
*Ccna2 (***NM_053702.3**)	5'-CTTTTAGTGCCGCTGTCTCTTT-3'	5'-GCCCGCATACTGTTAGTGATGT-3'
*Myc (***NM_012603.2**)	5'-ACCCAACATCAGCGGTCG-3'	5'-CGTGACTGTCGGGTTTTCCA-3'
*Ccnd1 (***NM_171992.4**)	5'-AAAATGCCAGAGGCGGATGA-3'	5'-GAAAGTGCGTTGTGCGGTAG-3'
*GAPDH (***NM_017008.4**)	5'-CACGGCAAGTTCAACGGCACAGTCA-3'	5'-GTGAAGACGCCAGTAGACTCCACGAC-3'

### Proliferation assays

MTT assay was used to measure the cell viability of BRL-3A cells. Briefly, after 0.02 mL of 5mg/ml MTT (Sigma, USA) was added to each well, the cells were incubated at 37°C for 4 h, then 0.15 mL of dimethylsulfoxide (DMSO) (Sigma, USA) was added to each well and the wells were gently shaken for 10 min at room temperature. The absorbance was measured at 490 nm by Biotek Reader (ELx800, USA). Proliferation measurement was performed by counting live cells in haemocytometer chamber after trypan blue staining. 1×10^5^ cells were seeded into 24-well plates and transfected with siRNA at a final concentration of 50 nM; while the transduced cells (over-expression C9orf116 cells) were seeded into 24-well plates at a density of 1×10^5^ cells/well. Cells were cultured during either: 24, 48 and 72h. Cells were trypsinized and re-suspended in 1 mL of fresh medium, stained with trypan blue during 5 minutes and living cells counted using a haemocytometer chamber.

### Cell apoptosis assay

To assess the development of apoptosis induced by C9orf116, cell apoptosis was evaluated by flow cytometry using the Annexin V PE Apoptosis kit (BD Pharmingen, USA). 1×105 cells were seeded into 24-well plates and transfected with siRNA at a final concentration of 50 nM; while the transduced cells (over-expression C9orf116 cells) were seeded into 24-well plates at a density of 1 × 10^5^ cells/well. Cells were harvested at 48h post-transfection. Then, cells were washed with cold PBS and resuspended in 100 μl 1 × binding buffer, followed by addition of 5 μl Annexin V-PE and 5 μl 7-AAD. The cells were incubated for 10 min at room temperature in the dark. Finally, 400 μl 1 × binding buffer were added to the cells, which were analyzed by flow cytometry.

### Cell cycle analysis

Cell cycle was analyzed using flow cytometry. 1×10^5^ cells were seeded into 24-well plates and transfected with siRNA at a final concentration of 50 nM; while the transduced cells (over-expression C9orf116 cells) were seeded into 24-well plates at a density of 1×10^5^ cells/well. Cells were harvested at 48h post-transfection. After being harvested, cells were first washed in cold PBS and then fixed in 70% alcohol at -20°C for 12 h at least. The fixed cells were washed in cold PBS for 3 times and incubated in 1 mL of PBS solution with 50 μg of propidium iodide (PI, Sigma, USA) and 100 μg of RNase A (Sigma, USA) for 30 min at 37°C. Samples were then analyzed for DNA content by FACSCan.

### EdU incorporation assay

EdU incorporation assay was carried out using Cell-Light EdU imaging detecting kit according to the manufacturer’s instructions (RiboBio). EdU is a thymidine analog whose incorporation can be used to label cells undergoing DNA replication [17]. Briefly, the cells were firstly treated with 50 μmol/L of EdU for 2 h at 37°C. After being fixed with 4% paraformaldehyde for 30 min, the cells were treated with 0.5% Triton X-100 for 10 min and washed with PBS three times. Then, the cells were exposed to 100 μL of 1×Apollo® reaction cocktail for 30 min and incubated with 5 μg/mL of Hoechst 33342 to stain the cell nuclei for 30 min. Images were captured by a fluorescent microscope.

### Western blot analysis

Cells samples were homogenized in RIPA lysis buffer (50 mM Tris, 150 mM NaCl, 1% Triton X-100, 1% sodium deoxycholate, 0.1% SDS) containing proteinase inhibitors (1 mM phenylmethylsulfonyl fluoride, 2 μg/ml aprotinin, and 2μg /ml leupeptin). Protein concentrations were determined using a BCA Protein Assay Kit (Beyotime biotechnology). Then, the cell lysates were boiled in SDS sample buffer for 5 min. Equal amount of protein per sample was separated by SDS-polyacrylamide gel electrophoresis and transferred to nitrocellulose membranes (GE Healthcare). The membranes were first blocked with 5% non-fat milk in tris-buffered saline (TBS) containing 0.1% tween-20 (TBS-T) and subsequently incubated with rabbit anti-C9orf116 (Our lab, 1: 1,000), rabbit anti-CCNA2 (Boster, 1: 1,000), rabbit anti-MYC (Boster, 1: 1,000), rabbit anti-CCND1 (Boster, 1: 1,000) and rabbit anti-phospho-histone H3 (Ser10) (CST, 1:1000) overnight at 4°C. Then the membrane was further incubated with horseradish peroxidase (HRP)-conjugated secondary antibodies goat anti-rabbit IgG (Sigma, 1:5,000). Finally, protein band was visualized with Amersham enhanced chemiluminescence (ECL) substrates (Western Lightning® Plus-ECL). The band density was measured using ImageQuant TL software. β-actin (sigma, 1:1,000) served as an internal reference.

### Statistical analysis

All data were described as means ± SD and experiments were performed three times and three replications per group. Data were analyzed by Student’s t test. A *P* value of less than 0.05 was considered to be statistically significant, *P*-value of less than 0.01 was considered to be statistically extremely significant.

## Results

### The effect of siRNAs on C9orf116 expression

**Fig 1:**
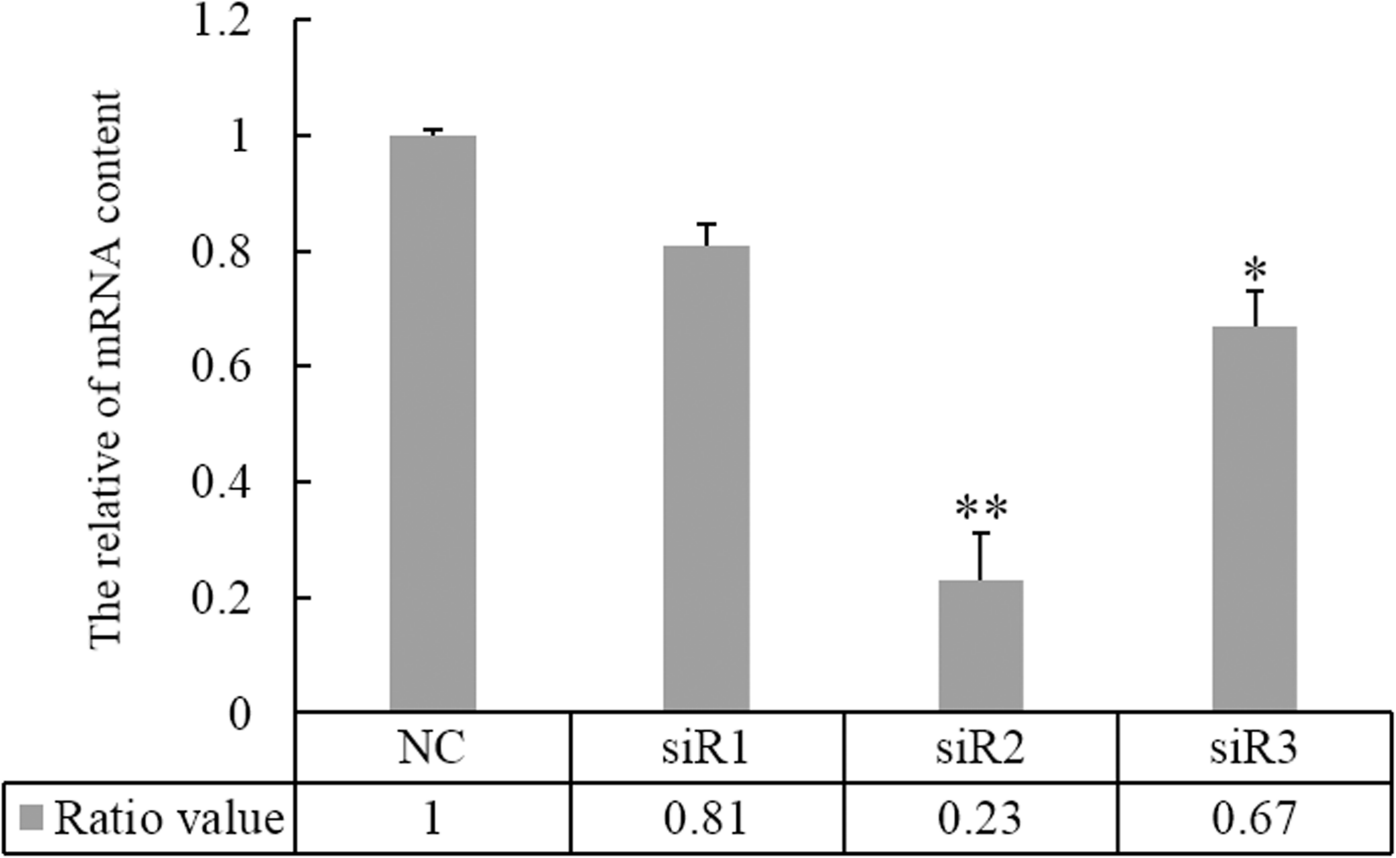
The effect of C9orf116 siRNAs on the mRNA expression level of C9orf116. NC. Negative siRNA; siR1, siR2 and siR3 represented cells treated with C9orf116 siRNA 1, siRNA 2 and siRNA 3, respectively. The experiments were performed three times and three replicates in each one, the data were shown as the means ± SD, *P<0.05, **P<0.01.

We designed three siRNAs based on C9orf116 sequence, to select the most effective of siRNA on C9orf116 expression, quantity RT-PCR was used to determine the effect of different siRNAs on the expression level of C9orf116. The results showed that siR2 was the most effective among the three siRNAs. The C9orf116 mRNA level was reduced to 23 ± 5.1% in siR2 group when compared with NC, whereas the C9orf116 mRNA level was only reduced to 67 ± 2.9% in siR3 and to 81 ± 1.5% in siR1 group (Fig 1). Statistical analysis revealed that the mRNA level of C9orf116 was significantly declined in BRL-3A cells transfected with siR2 (*p*<0.01 vs control) and siR3 (*p*<0.05) at post-48h, but no significant difference was found between siR1 and NC. Therefore, we performed follow-up research with siR2.

### Preparation of virus and identification of C9orf116 over-expression

**Fig 2:**
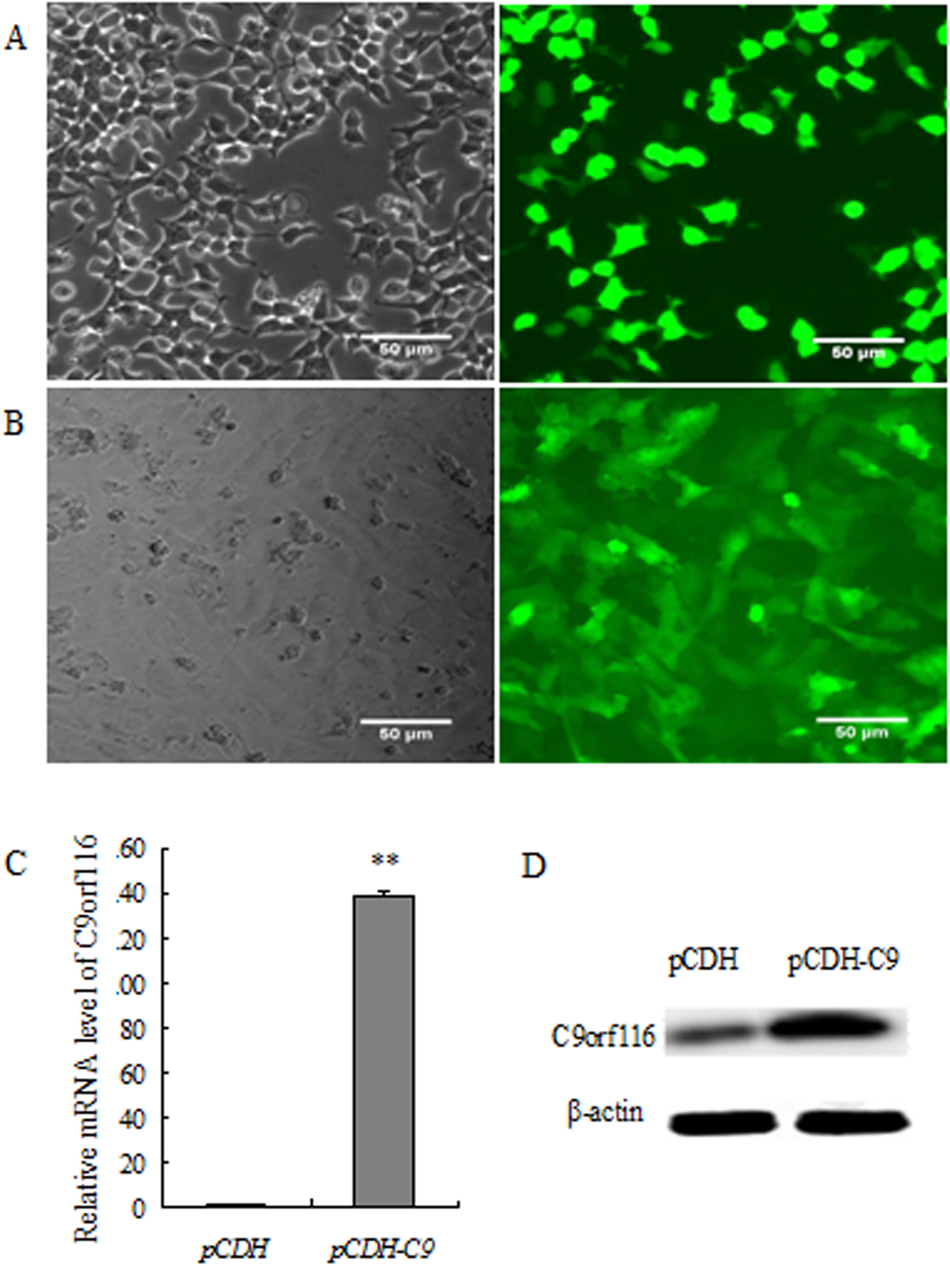
Preparation of virus and identification of C9orf116 over-expression. (A) Viral plasmid was transfected into HEK293T cells, the left figure was bright field, the right picture was fluorescence (B) BRL-3A cells were infected with virus particles, the left figure was bright field, the right picture was fluorescence (C/D) The change expression of C9orf116 at both mRNA(C) and protein(D) levels in BRL-3A. The experiments were performed three times and three replicates in each one. The data were shown as the means ± SD. Representative images were shown ** Indicates *p* <0.01.

About 90% of the cells exhibited high-intensity fluorescence at 24 h after the plasmid pCDH-C9orf116 (pCDH-C9) was transfected into HEK293T cells, indicating a successful viral packaging (Fig 2A). The virus was filtered and concentrated virus titer was 1.5 × 10^8^TU/ml, and used for subsequent experiments. About 95% of BRL-3A cells exhibited high-intensity fluorescence at 72h after lentivirus infection (Fig 2B). RT-PCR and Western blot analysis indicated that the expression of C9orf116 was significantly increased in BRL-3A-pCDH-C9orf116 group cells when compared with the pCDH group (Fig 2C and 2D) (*p* <0.01).

### Effect of C9orf116 on BRL-3A cell proliferation

**Fig 3:**
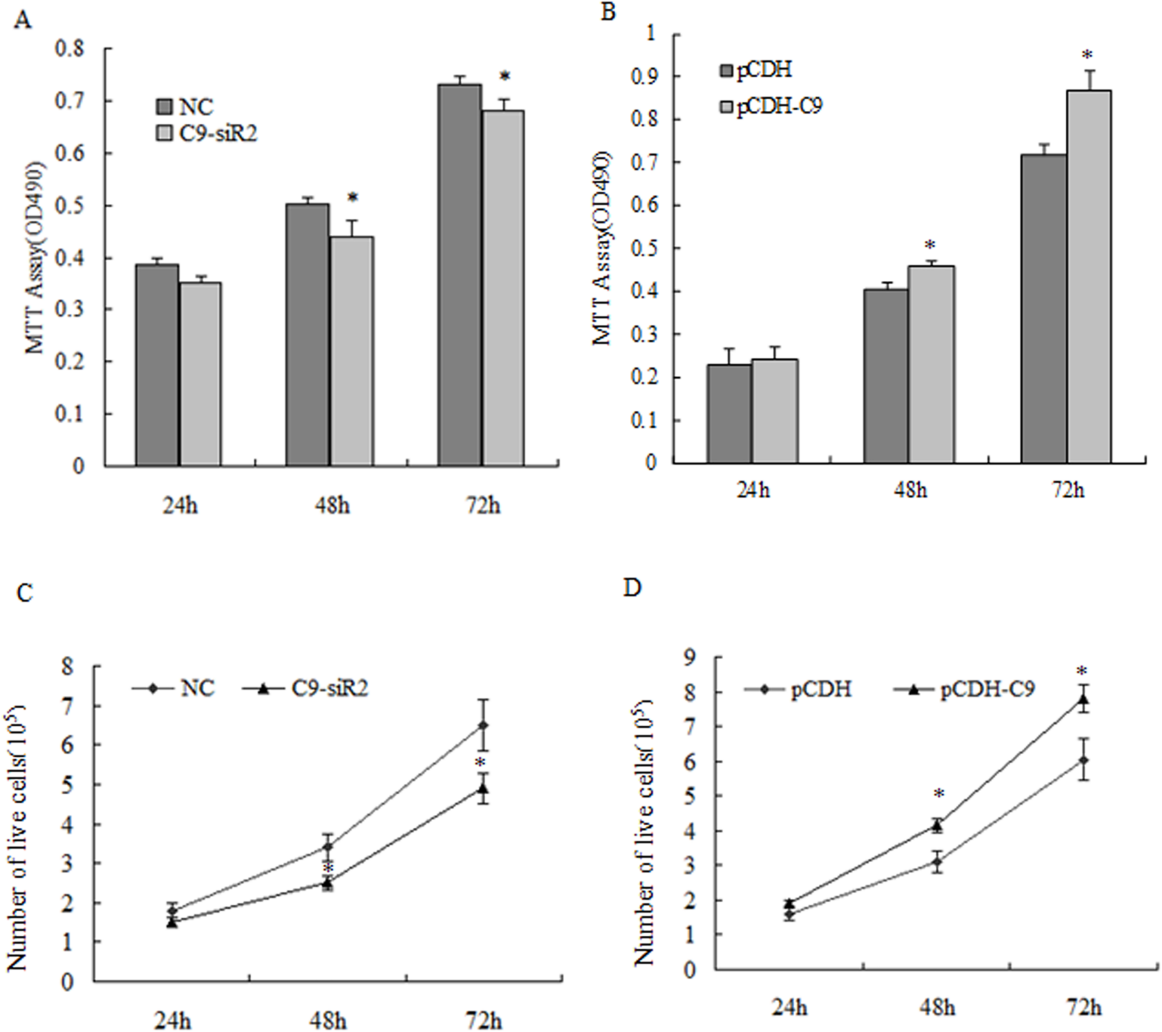
Effect of C9orf116 on BRL-3A cell proliferation. (A) The cell viability was assessed at 24, 48 and 72 h after transfected with siRNA and control by MTT assay. (B) The cell viability was assessed at 24, 48 and 72 h in C9orf116 over-expression cells and control by MTT assay. (C) Proliferation measurement by counting live cells in haemocytometer chamber after trypan blue staining, the number of live cells was counted at 24, 48 and 72 h after transfected with siRNA and control. (D) The number of live cells was counted at 24, 48 and 72 h in C9orf116 over-expression cells and control. NC, C9-siR2 represents the cells treated with negative siRNA, siR2-C9orf116, respectively; pCDH, pCDH-C9 represents the cells treated with Empty vector (pCDH), pCDH-C9orf116, respectively. The above results were presented as means ± SD. The experiments were performed three times and three replications per group. * Indicates *p* <0.05.

To assess the cell proliferation effect of C9orf116 on BRL-3A cells, the cells viability and live cells counting were measured at 24, 48 and 72 h after C9orf116 knockdown and over-expression in BRL-3A cells. The results showed that the cell viability began to decline at 24 h and was significantly lower than that of the NC group at 48 h and 72 h in siR-transfected groups (Fig 3A) (*p*<0.05). The cell viability in the over-expression group was significantly increased at 48 and 72 h compared to the pCDH group. No significant difference was observed at 24 h, though it was still higher than that of the pCDH group (Fig 3B). The number of live cells in siR-transfected groups was about 25% smaller than that in the control group at 48 and 72 h(Fig 3C) (*p*<0.05). On the other hand, the number of live cells in overexpression group was about 30% higher than that in the control group at 48 and 72 h(Fig 3D) (*p*<0.05). No significant difference in cell count was observed in BRL-3A cells knockdown and over-expression of C9orf116 at 24 h. Therefore, we performed follow-up research at 48 h.

### Effect of C9orf116 on BRL-3A apoptosis and cell cycle

**Fig 4:**
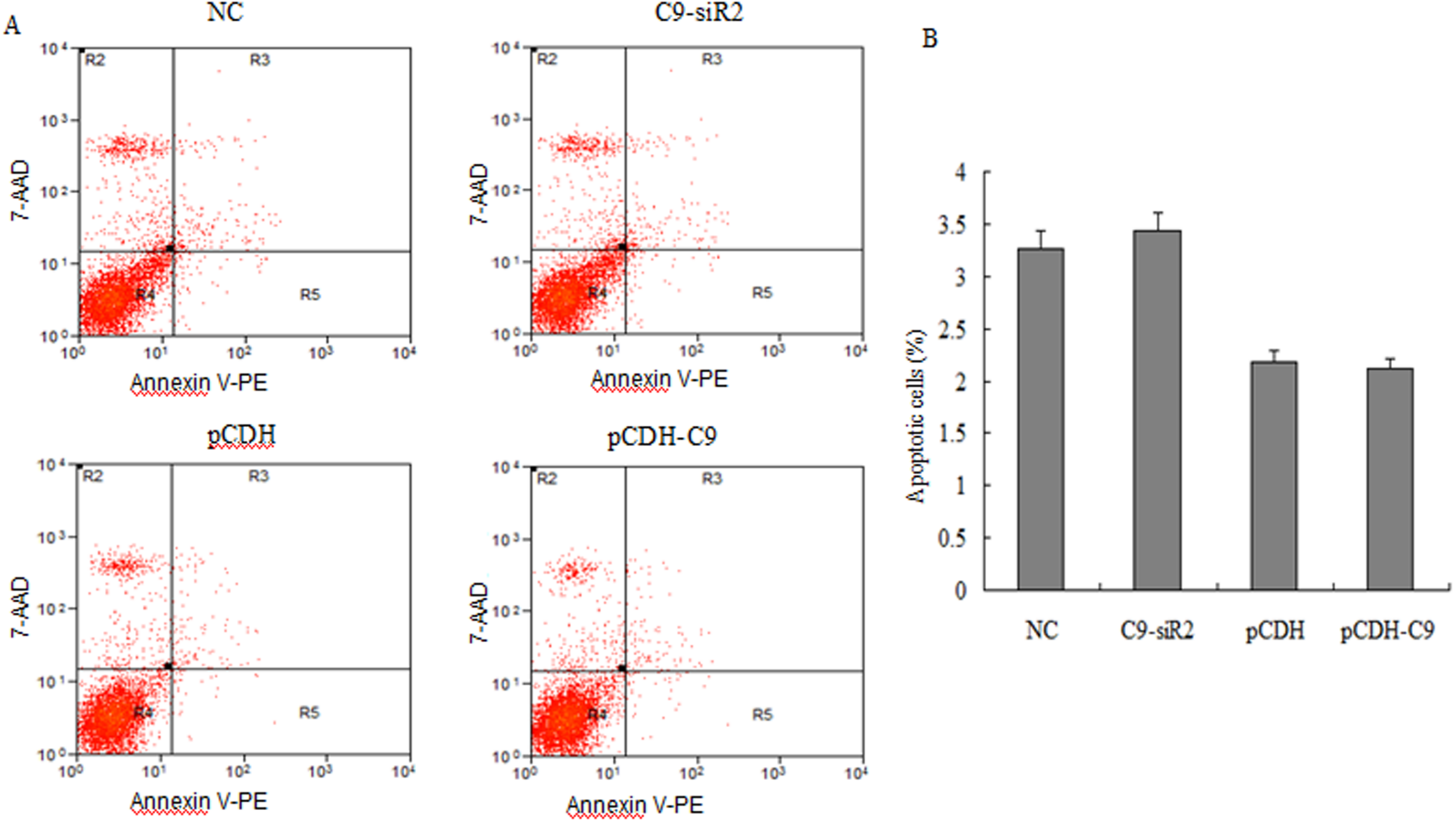
Effect of C9orf116 on apoptosis of BRL-3A cells. (A) Representative graphs obtained by flow cytometric analysis after double-staining with Annexin V-PE/7-AAD. (B) The percentage of late and early apoptotic cells were summed to give the total number of apoptotic cells. The experiments were performed three times and three replicates in each one. The data were shown as the means ± SD.

**Fig 5:**
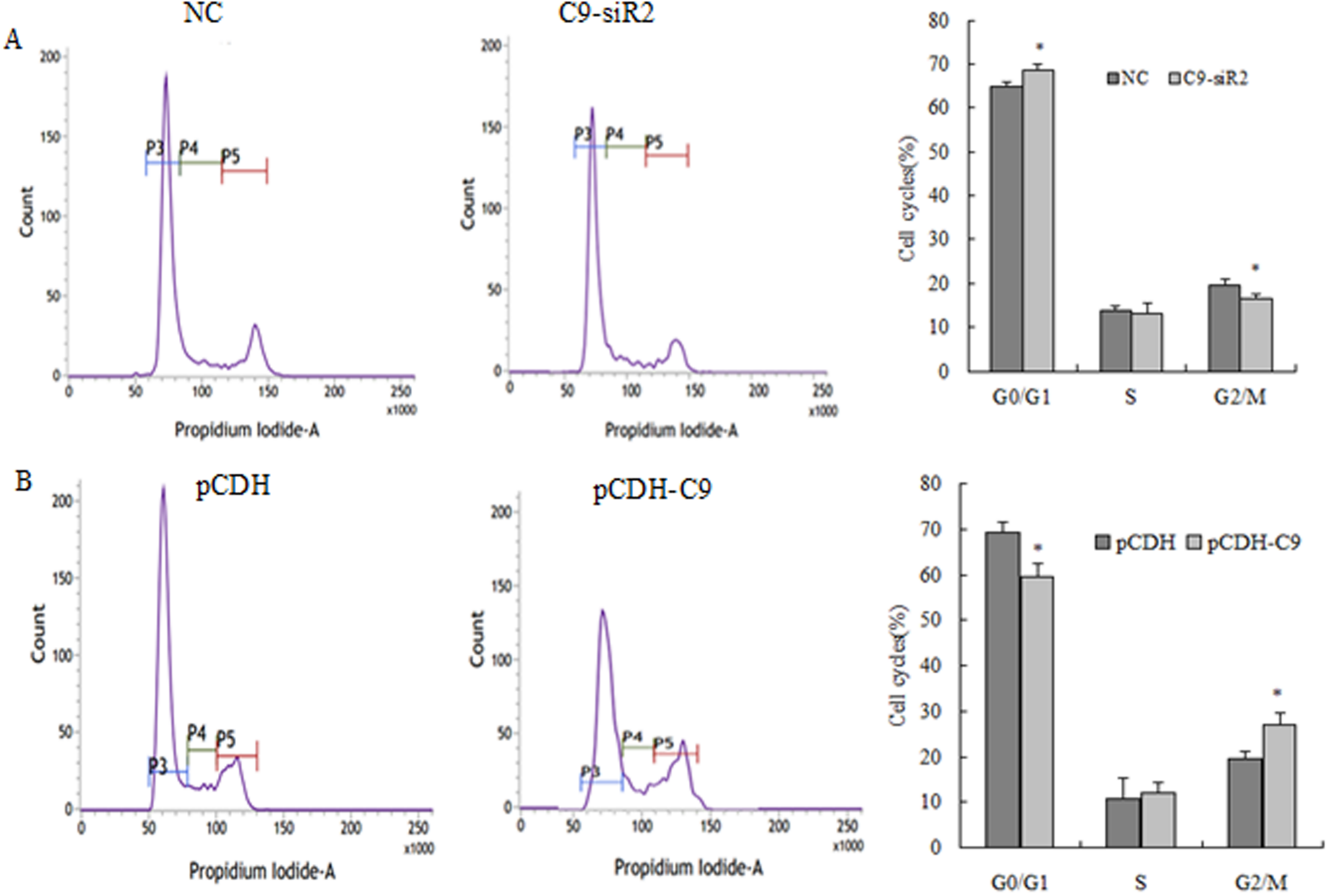
Effect of C9orf116 on BRL-3A cell cycle distribution. (A) Cell cycle distribution was assessed at 48 h after transfected with siRNA and control by flow cytometry. (B) Cell cycle distribution was assessed at 48 h in C9orf116 over-expression cells and control by flow cytometry. The experiments were performed three times and three replicates in each one. The data were shown as the means ± SD. Representative images were shown * Indicates *p* <0.05.

To explore how C9orf116 affected cell proliferation, cell apoptosis and cell cycle analysis by flow cytometry were carried out. We found that down-regulation of C9orf116 expression or up-regulation of C9orf116 expression had little effect on BRL-3A cell apoptosis (Fig 4A and 4B). Thus, we hypothesized that C9orf116 may contribute to cell cycle regulation to regulate BRL-3A cell proliferation. Indeed, following C9orf116 knockdown and over-expression, the result showed that 69.58 ± 1.27% of C9-siR2 cells were at the G0/G1-phase with 13.38 ± 1.31% cells at G2/M-phase; whereas the control group only has 62.75 ± 1.15% cells at the G0/G1-phase cells with 19.91 ± 1.12% cells at G2/M-phase (Fig 5A). At the same time, the cell population of G0/G1-phase cells in pCDH-C9 and pCDH group was 59.56 ± 2.52% and 69.36 ± 2.13%, respectively and the percentage of G2/M-phase cells was 26.96 ± 3.52% and 19.63 ± 2.93% (Fig 5B), respectively. Thus, C9orf116 appears to modulate BRL-3A cell proliferation by controlling the cell cycle without affecting cell apoptosis.

### Effect of C9orf116 on proliferation-related genes in BRL-3A cell

**Fig 6:**
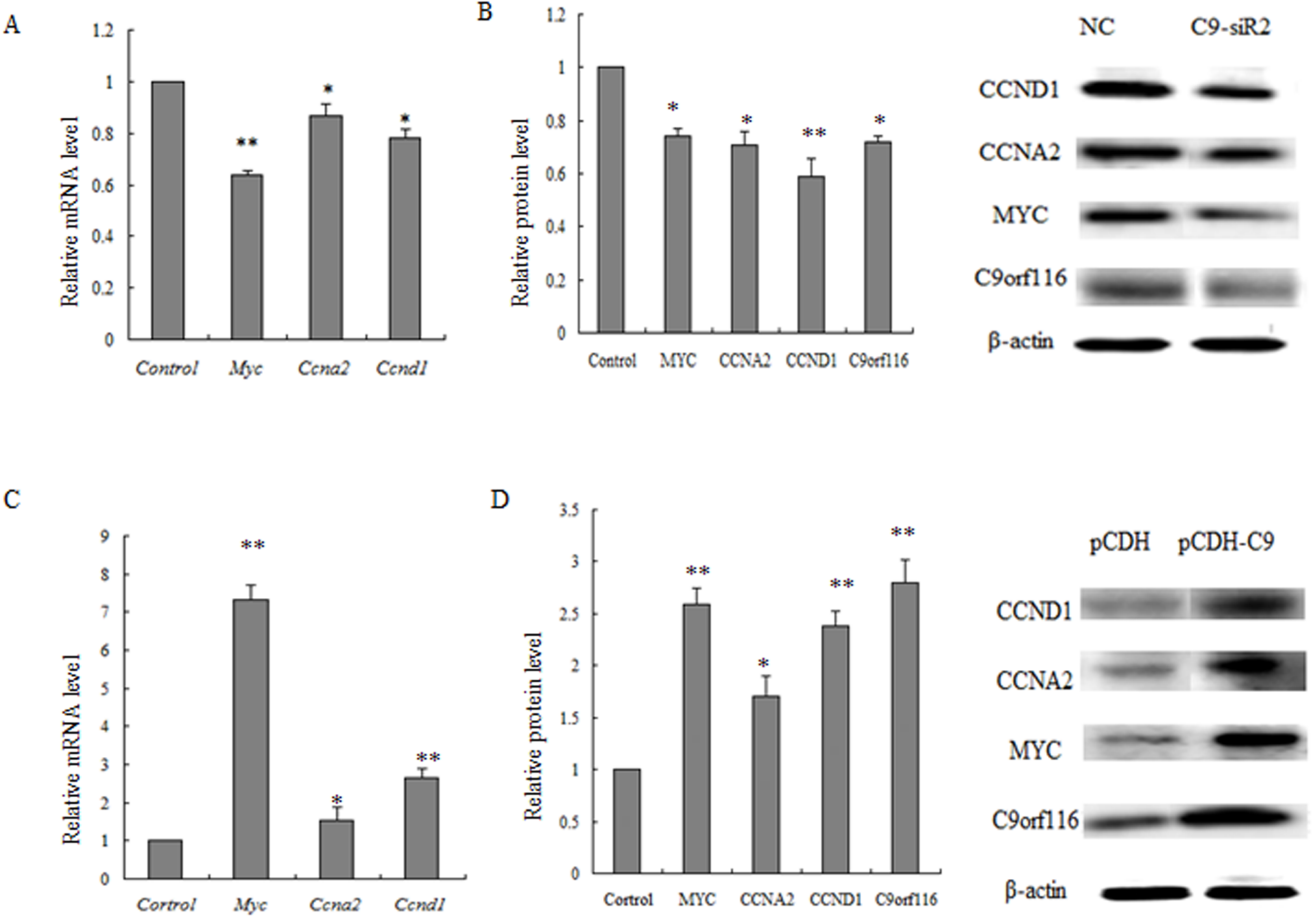
Effect C9orf116 on the expression of cell proliferation related genes. (A) The expression of cell proliferation related genes in C9-siR2 group at the mRNA level by qRT-PCR analysis. (B) The expression of cell proliferation related genes in C9-siR2 group at the protein level by western blot analysis. (C) The expression of cell proliferation related genes in pCDH-C9 group at the mRNA level by qRT-PCR analysis. (D) The expression of cell proliferation related genes in pCDH-C9 group at the protein level by western blot analysis. The experiments were performed three times and three replicates in each one, the data were shown as the means ± SD. Representative images were shown *P<0.05, **P<0.01.

RT-PCR and Western Blot were used to detect the expression changes of proliferation-related genes after interference and over-expression of C9orf116 in BRL-3A cell. The results showed that the expression of cell proliferation related genes including CCNA2, CCND1 and MYC was significantly lower in the C9-siR2 infected cells than that of control group (Fig 6A and 6B). Meanwhile, the expressions of cell proliferation related genes CCNA2, CCND1 and MYC were significantly higher in cells over-expressing C9orf116 than that of control group (Fig 6C and 6D).

### Effect of C9orf116 on mitotic entry

**Fig 7:**
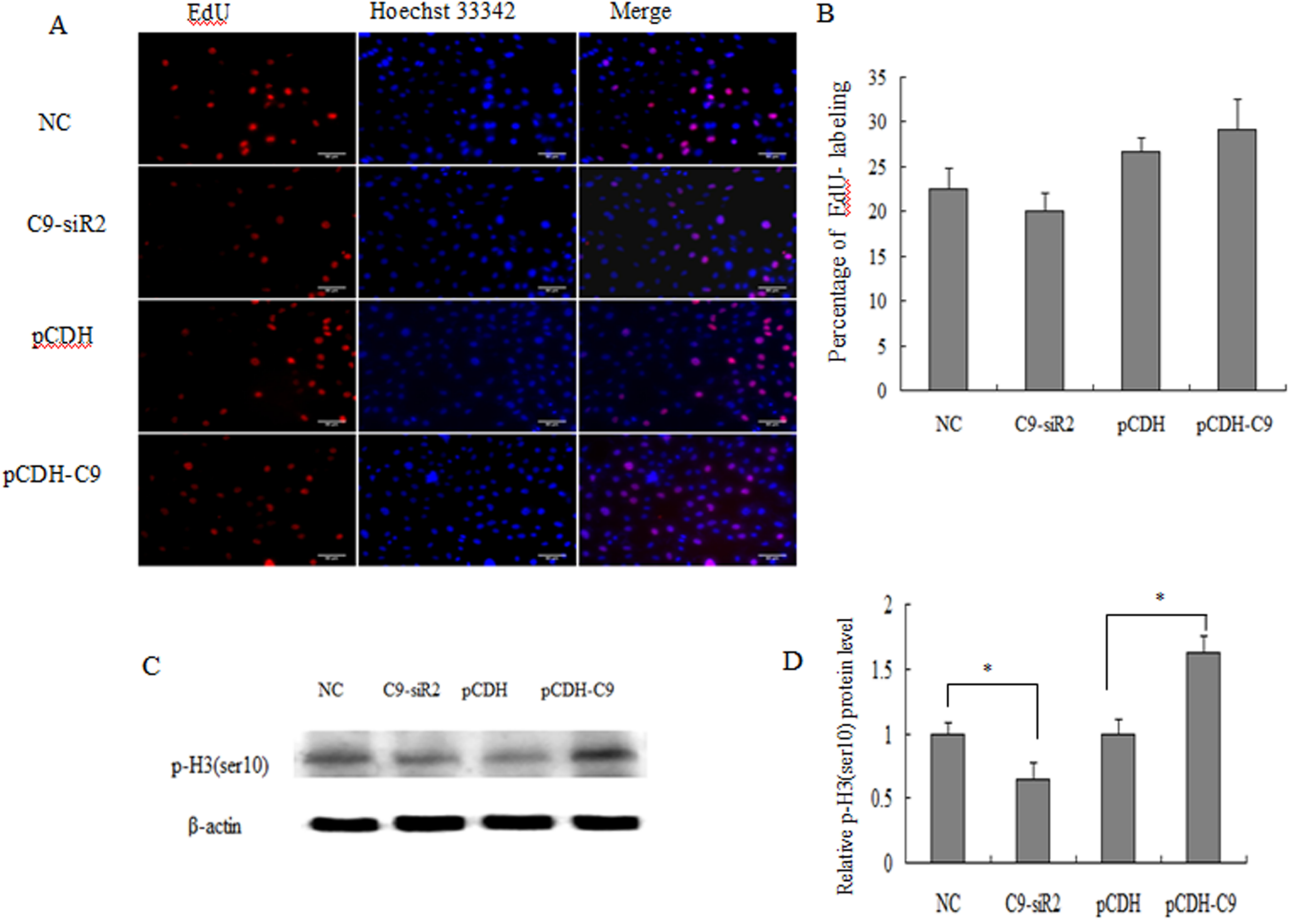
Effect of C9orf116 on mitotic entry (A) EdU incorporation assays. The Click-it reaction revealed EdU staining (red). Cell nuclei were stained with Hoechst 33342 (blue). (B) The percentage of EdU-positive cells was quantified. (C) The expression of p-H3 (Ser10) was evaluated by Western blot analysis in C9orf116 knockdown and overexpression BRL-3A cells compared to their control, respectively. (D) The expression of p-H3 (Ser10) was quantified. The experiments were performed three times and three replicates in each one. The data were shown as the means ± SD. Representative images were shown *P<0.05, **P<0.01.

To determine more precisely which phase of the cell cycle was affected by C9orf116 expression, EdU incorporation was used for the analysis of S phase synthesis. Cells were labeled with EdU, and EdU mitotic cells were captured by a fluorescent microscope. C9-siR2 and pCDH-C9 groups had similar EdU incorporation rates (Fig 7A and 7B), which indicated that C9orf116 knockdown or over-expression had little effect on S-phase DNA synthesis. The mitotic index (the percentage of cells in the M phase) was performed using phosphorylated histone H3 (Ser10; p-H3 [Ser10]) expression by western blot analysis. Because p-H3 (Ser10) is a marker of mitosis and association with mitotic chromatin condensation in M phase of the cell cycle [18–19]. Western blot analyses showed that down-regulation of C9orf116 expression resulted in a significant decrease of p-H3 (Ser10) compared to control (Fig 7C and 7D), and upregulation of C9orf116 expression increased p-H3 (Ser10) expression compared to control (Fig 7C and 7D). The results suggested that C9orf116 knockdown or over-expression affected the percentage of M phase, which indicated that C9orf116 over-expression accelerate mitotic entry.

## Discussions

The liver has a variety of physiological functions. In order to further study the molecular mechanism of liver regeneration, we performed genomics and proteomics studies of liver regeneration and found that C9orf116 was highly expressed in the regenerated liver [11, 12]. Therefore, we hypothesized that it might be related to liver cells proliferation in LR. To further confirm the role of C9orf116 in normal liver cells, we used BRL3A cells as models to study the function of C9orf116 in vitro [20–22]. RNA interference has been developed as an important means to study gene function. Lentiviral vectors have little immune response, and are able to infect non-dividing cells with a high transfection efficiency, therefore, they are also often used to study the function of genes [14, 15, 23–26]. In this study, we used lentiviral vector for over-expression of C9orf116 and siRNA to knockdown the expression of C9orf116, and further analyzed its possible role in BRL-3A cells.

We found that C9orf116 mediated BRL-3A cell proliferation via regulating cell cycle progression, and had no effect on apoptosis. Following C9orf116 knockdown or over-expression, we found that BRL-3A cells decreased or accumulated in G2/M stages, and cell proliferation-related genes MYC, CCND1 and CCNA2 were down or up-regulated.

c-Fos, c-Jun and c-Myc are immediate-early genes in liver regeneration, they induce the transition of liver cells from the G0 phase to G1 phase regulate start-up phase of the cell cycle, and initiate liver regeneration process [27–29]. As an oncogene, c-Myc can promote cell proliferation. Some studies showed that increased expression of c-Myc was associated with an increase in expression of c-Myc-regulated genes such as cyclin D1 and cyclin E, whereas c-Myc silencing reduced expression of cyclin D1 and cyclin E and delayed the function of epithelial repair [30–32]. Other researches showed that c-Myc was an important driver of replication in the two most commonly employed rat β-cell lines [33]. Cyclin D1 was an important sign to detect liver cells entering into the G1 phase [34], but also an important target protein with a variety of mitogen [35, 36]. Activator protein 1 (AP-1) has some binding sites with the promoter region of cyclin D1.A combination of c-Fos either or c-Jun would induce the expression of cyclin D1 in mRNA level [37]. Recently, some studies have shown that cyclin D1 is significantly up-regulated in liver cells after PH, and regulates cells going through the G1-phase checkpoint, and promotes cell proliferation [38, 39]. Cyclin A2, which is particular in the cyclin family, activates two different cyclin-dependent kinases (CDKs) and promotes both G1/S and G2/M phase transitions [40]. Studies showed that Cyclin A2 promotes hepatocyte cell cycle progression and liver regeneration [41, 42]. In this study, our research showed that the expression of cell proliferation related genes including CCNA2, CCND1 and MYC was significantly lower than that of NC group both in mRNA and protein level after interference C9orf116 with C9-siR2. At the same time, the expression of cell proliferation related genes CCNA2, CCND1 and MYC was significantly higher than that of control group both at mRNA and protein level after C9orf116 over-expression with pCDH-C9. So we inferred that C9orf116 may activate CCNA2, CCND1 and MYC to promote cell proliferation in rat liver cell line BRL-3A.

To determine more precisely which phase of the cell cycle was affected by C9orf116 expression, EdU incorporation and phosphorylated histone H3 (Ser10; p-H3 [Ser10]) expression were used to determine S phase DNA synthesis and M phase enter. EdU is a thymidine analog whose incorporation can be used to label cells undergoing DNA replication [17]. Phosphorylated histone H3 (Ser10; p-H3 [Ser10]) is a marker of mitosis and association with mitotic chromatin condensation in the M phase of the cell cycle [18, 19]. Recently, p-H3 (Ser10) is used as an indicator of mitosis in many studies [43–46]. The results showed that C9-siR2 and pCDH-C9 groups had similar EdU incorporation rates following C9orf116 knockdown or over-expression. While C9orf116 knockdown decreased, and C9orf116 over-expression promoted, p-H3 (Ser10) expression, which suggested that M phase but not G2 and S phase is modulated by C9orf116 in BRL-3A cells.

In conclusion, the study demonstrates that C9orf116 could promote cell proliferation by modulating cell cycle transition and the expression of key genes CCNA2, CCND1 and MYC in BRL-3A cells. In the future, we will investigate the mechanism of C9orf116 in regulating cell proliferation in vitro and in vivo, and we will focus on its signaling pathways and the precise regulation of C9orf116 by protein modification, such as phosphorylation and methylation.
